# Impact of biannual azithromycin on weight-for-age z-score among infants in the AVENIR cluster-randomised trial

**DOI:** 10.1136/bmjpo-2025-004037

**Published:** 2025-12-11

**Authors:** Brittany Peterson, Ahmed Arzika, Ramatou Maliki, Amza Abdou, Bawa Aichatou, Ismael M Bello, Diallo Beidi, Nasser Galo, Nasser Harouna, Alio M Karamba, Sani Mahamadou, Moustapha Abarchi, Almou Ibrahim, Elodie Lebas, Zijun Liu, Carolyn Brandt, Emily Colby, Catherine Oldenburg, Travis Porco, Benjamin F Arnold, Thomas M Lietman, Kieran S O’brien

**Affiliations:** 1 F.I. Proctor Foundation, University of California San Francisco, San Francisco, California, USA; 2 Department of Epidemiology and Biostatistics, University of California San Francisco, San Francisco, California, USA; 3 Centre de Recherche et Interventions en Santé Publique, Birni N’Gaoure, Niger; 4 Programme Nationale de Santé Oculaire, Niamey, Niger; 5 Department of Opthalmology, University of California San Francisco, San Francisco, California, USA; 6 Institute for Global Health Sciences, University of California, San Francisco, San Francisco, California, USA

**Keywords:** Child Health, Infant, Epidemiology

## Abstract

**Background:**

The AVENIR cluster-randomised trial evaluated whether biannual mass drug administration (MDA) of azithromycin improved childhood mortality among infants in Niger, a high-mortality, high-malnutrition setting.

**Methods:**

Weight-for-age z-score (WAZ) was a prespecified secondary outcome of the trial. In this analysis, 2880 communities in the Dosso and Tahoua regions and 51 687 infants with two sequential weight measurements were included. Weight was assessed at baseline and 6 months post treatment, and WAZ was calculated using WHO standards. To include two subsequent measures of WAZ, infants at baseline were 1–5 months old. Linear mixed-effects models compared mean WAZ between azithromycin and placebo arms, with additional analyses examining community-level means, categorical underweight outcomes and predefined subgroups.

**Results:**

At follow-up, mean WAZ was −1.39 in the azithromycin arm and −1.38 in the placebo arm, with no detectable difference (mean difference 0.00, 95% CI −0.05 to 0.04, p=0.94). Odds of moderate-to-severe or severe underweight also did not differ between groups. Subgroup analyses by baseline nutritional status, age, sex, community size and distance to health centres showed consistent null effects, though a small, non-significant improvement in WAZ was observed in infants treated during the rainy season compared with the dry season.

**Conclusion:**

These findings align with prior large-scale trials that demonstrated mortality benefits but no sustained effects of azithromycin on growth. Taken together, the results suggest that while azithromycin MDA reduces childhood mortality, it is unlikely to provide medium-term improvements in weight gain, highlighting the need for complementary nutritional interventions to address undernutrition in similar settings.

**WHAT IS ALREADY KNOWN ON THIS TOPIC**

- Several trials have demonstrated a 14–18% decrease in child mortality following azithromycin mass drug administration (MDA) to children 1–59 months. Prior studies assessing the impact of MDA have not consistently demonstrated effects on nutritional outcomes such as growth, which may be due to many of these trials having limited statistical power due to small sample sizes.

**WHAT THIS STUDY ADDS**

- The AVENIR trial provides a larger sample size than previous trials which allows for more power to detect differences in weight-for-age z-score.

**HOW THIS STUDY MIGHT AFFECT RESEARCH, PRACTICE OR POLICY**

- When considered alongside other large community-based trials in sub-Saharan Africa that have similarly reported no sustained effects of azithromycin MDA on anthropometric outcomes, our findings contribute to a growing body of evidence indicating that antibiotic distribution, while effective at reducing mortality, is unlikely to meaningfully improve child growth in the absence of additional interventions.

## Background

 Biannual mass drug administration (MDA) of antibiotics has proven to be effective in decreasing under-5 mortality in West Africa. Several recent trials have demonstrated a 14–18% decrease in child mortality with biannual azithromycin distribution to children 1–59 months.^1^,^3^ Malnutrition contributes to nearly half of all under-5 deaths globally by increasing vulnerability to infectious diseases and limiting recovery from illness.^4^ Because MDA programmes often target high-mortality settings where malnutrition is prevalent, understanding their impact on nutritional status is critical both for interpreting mortality effects and for identifying potential co-benefits of antibiotic distribution.

Children who are malnourished have an increased risk of death from infectious diseases.^5 6^ The interaction between infectious disease and malnutrition is complex: infection can impair nutritional status through mechanisms such as malabsorption, increased nutrient demands and appetite suppression.^7 8^ Conversely, undernutrition weakens the immune system and increases susceptibility to infection.^7 8^ An analysis of causes of death from children under 5 in several Low- and middle-income countries (LMICs) in Africa and South Asia assessed by an expert panel showed that malnutrition was a causal or significant factor in 39.5% of deaths, with 89.1% of those also involving infectious diseases.^6^ Antibiotics are frequently used in the treatment and management of severe acute malnutrition, especially in clinical settings.^9 10^ However, little is known about the potential for administration of antibiotics via MDA to improve nutritional outcomes at the community level.

The hypothesis that reducing infectious burden might support growth has prompted investigation into the role of azithromycin MDA, but studies in several settings have not found evidence of this. In Burkina Faso, a 2021 trial found a short-term increase in child weight gain 14 days after a single dose of azithromycin but no lasting effect at 6 months, while two other trials in younger infants found no effect on growth at 6 months.^11^,^13^ Similarly, a community-level MDA study in The Gambia found no significant differences in stunting, wasting or underweight between children receiving one versus three annual rounds of azithromycin.^14^ In another trial in Niger assessing biannual azithromycin MDA in children 1–59 months compared with placebo, no improvements were found in weight or height over time compared with placebo.^15^ While prior studies assessing the impact of MDA have not consistently demonstrated effects on nutritional outcomes such as growth, many of these trials had limited statistical power due to small sample sizes. The AVENIR trial, with one of the largest sample sizes to date, provides a unique opportunity to assess whether azithromycin MDA impacts weight-for-age in a real-world, community-based setting.

The purpose of this study is to leverage the scale of the data collected from the AVENIR trial to assess the relationship between azithromycin MDA and weight-for-age z-score (WAZ) in infants. We hypothesise that infants receiving antibiotic treatment will have better weight-for-age outcomes than infants receiving placebo. Given the trial’s large sample size, this analysis provides an opportunity to determine whether antibiotic MDA has secondary benefits on infant growth beyond its established effect on mortality.

## Methods

### Trial design, setting and population

This is a pre-specified secondary analysis of the AVENIR cluster-randomised, response-adaptive trial. AVENIR randomised communities to one of three arms: (1) azithromycin to children 1–59 months, (2) azithromycin to infants 1–11 months with placebo to children 12–59 months and (3) placebo to all children aged 1–59 months. The trial protocol and primary results are published elsewhere.^3 16^ AVENIR included rural and peri-urban communities with a population size between 250 and 2499 located in the Dosso and Tahoua regions of Niger. Children were eligible for treatment if they weighed at least 3 kg, had no known allergy to macrolides and had guardian consent. This analysis included only infants who had two sequential weight measurements which restricted inclusion to those who were 1–5 months old at time of baseline data collection.

### Patient and public involvement

Neither patients nor the public were involved in the design of this study. However, the Niger Ministry of Health contributed to the study’s design. Additionally, the Niger Ministry of Health, primary health care center (CSI) leaders, community health workers and community leaders were engaged in the recruitment, implementation and dissemination phases of the study.

### Data collection

Data were collected every 6 months during a rolling door-to-door household census (dataset).^17^ Trained census workers entered census data using a custom CommCare application (Dimagi, Cambridge, Massachusetts, USA). Data collected included demographic information for every child aged 1–59 months old, guardian and head of household. Vital status was tracked at each follow-up visit. Weight measurements were obtained for all infants aged 1–11 months eligible for treatment to calculate dosage, using a hanging scale (ADE M111600, GmbH & Co., Hamburg, Germany). Trained census workers recorded the child’s weight to the nearest 0.1 kg. Census workers were instructed to ensure the child wore only light clothing, verify the scale displayed ‘0.00’ before use and then place the child in the weighing harness to obtain the measurement. Children aged 12–59 months had their dose calculated using a height-based dosing pole which indicates doses that are based on height ranges, but does not indicate specific heights.

### Intervention

Children received either azithromycin (Pfizer, Inc., New York, New York, USA) or a visually identical placebo as a single oral dose of 20 mg/kg. For those aged 1–11 months, dosing was based on weight measured using a hanging scale as described above, while for children aged 12–59 months, dosage was determined using a height-based dosing pole. Weight was only used for dosage for the younger age group due to the logistic complication of using the hanging scale at community members’ homes, and therefore was only used for the younger age group as they are least likely to be able to stand for height measurement.

### Randomisation and masking

Communities were assigned to one of the three treatment groups through randomisation. During the third and fourth phases of the trial, a response-adaptive allocation approach was implemented for newly enrolled communities. This method aimed to improve the chances that communities would receive the more effective treatment, promoting equity while preserving statistical integrity. Further details on this adaptive strategy are provided in the primary results publication.^3^ The randomisation sequence was created by a single unmasked biostatistician, while all other team members, including data analysts, field staff, investigators and participants, remained masked. To support masking, both the active treatment and placebo were packaged identically.

### Statistical analysis

The sample size for AVENIR was based on the primary outcome, childhood mortality; thus, the sample size for this analysis was fixed at 2880 clusters. Accounting for clustering (ICC=0.104) and unequal cluster sizes, our fixed sample size yielded a minimum detectable effect of 0.059 WAZ units at 80% power (α=0.05). Infants were eligible for statistical analysis in the present study if they had two consecutive weights taken, one at baseline before the child’s first treatment and the other as the outcome of interest, 6 months after the first treatment. For the present analysis, the azithromycin treatment arms were combined as all infants 1–11 months received treatment in both azithromycin arms and the total analysis population included only infants 1–11 months old. The primary outcome was WAZ as a continuous variable at the second instance of capture. The ‘anthro’ package in R was used to calculate the WAZ.^18^,^20^ Measurements that were <−6 and >5 were removed as outliers based on WHO recommendations.^19^ Secondary outcomes included mean WAZ at the community level, as well as categorising child-level WAZ into undernutrition groups: severe underweight (WAZ <3) versus not severe underweight (WAZ >=3) and moderate or severe underweight (WAZ <2) versus not moderate or severe underweight (WAZ >=2).

Baseline demographic characteristics at the child and community levels were summarised for each arm. The primary analysis used a linear mixed-effects model to calculate the difference in mean WAZ values at second instance of weight capture by treatment arm, adjusting for baseline WAZ and accounting for clustering by including community as a random effect. A sensitivity analysis involved disaggregating the two azithromycin arms, comparing the azithromycin 1–59-month arm versus placebo and azithromycin 1–11-month arm versus placebo. Community-level differences in mean WAZ were compared using a linear regression model. All infants with at least one weight captured are included in this analysis, and all communities with at least two consecutive treatments were included.

Subgroup analyses looking at difference in mean WAZ values at the second instance of weight capture were done in the same method as the primary, including subgroups by baseline WAZ, sex, community size, age at baseline, distance to nearest primary health centre and season. Baseline WAZ was categorised as severe underweight (WAZ <−3), moderate underweight (WAZ >=−3 and <−2) and not underweight (WAZ >=−2). Community size was based on the median community size in the study population using census data, either less than the median or greater than or equal to the median. Baseline age was dichotomised: 1–5 months and 6–11 months. Season was based on the rainy season in Niger occurring July to September and dry season occurring October to June. Other secondary analyses used categorical measurements of WAZ for indicators of malnutrition and conducting two outcome comparisons: severe acute malnutrition (WAZ <3) versus no severe acute malnutrition (WAZ >=3) and moderate and severe malnutrition (WAZ <2) versus no moderate or severe malnutrition (WAZ >=2). A generalised linear mixed model with a logit link and binomial family function was used with a random effect for village and adjusting for mean baseline WAZ. All analyses reported effect estimates with their 95% CIs and a permutation p value based on 1000 resamplings of the community-level treatment assignments.

## Results

**Figure 1:**
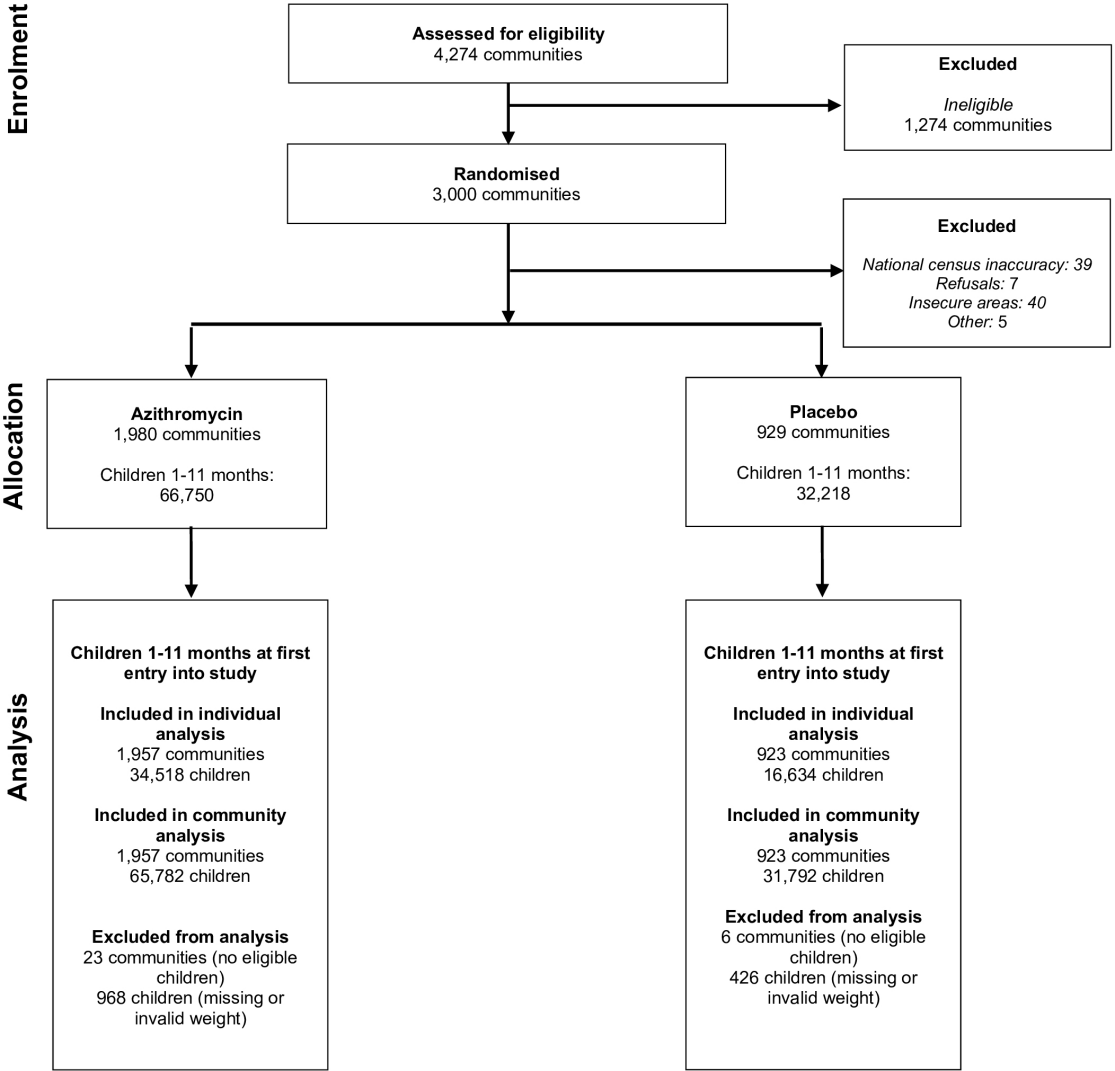
Participant flow diagram.

The trial was conducted from November 2020 to July 2023, enrolling 3000 communities and 98 968 infants aged 1–11 months over the 2-year duration. For the individual-level analysis, 2880 communities and 51 152 infants had weight measurements from at least two sequential rounds and were included (figure 1). Following WHO recommendations, 446 infants with a WAZ below −6 or above 5 for either the baseline or follow-up measurement were excluded.^19^ For the community-level analysis, 2880 communities and 97 574 infants had weight measurements from at least one round and were included. Demographic statistics were similar across arms (table 1). At baseline, mean WAZ was −0.80 (SD=1.5) in the azithromycin arm and −0.77 (SD=1.5) in the placebo arm. The proportion of infants moderately to severely underweight was 19.9% (n=6879) in the azithromycin arm and 19.5% (n=3243) in the placebo arm. The proportion of infants severely underweight was 7.5% (n=2605) in the azithromycin arm and 7.4% (n=1238) in the placebo arm.

**Table 1:** Community-level descriptive characteristics by arm of infants at their first census

	Azithro n=1957	Placebo n=923	Overall n=2880
Age in months, mean (SD)	3.5 (1.6)	3.5 (1.6)	3.5 (1.6)
Percent female, N (%)	17 377 (50.3)	8291 (49.8)	25 668 (50.2)
Mean child per community, mean (SD)	33.6 (0)	34.5 (0)	33.9 (0.4)
Population size per community, mean (SD)	705 (467)	669 (456)	703 (463)
WAZ, mean (SD)	−0.8 (1.5)	−0.8 (1.5)	−0.8 (1.5)
Proportion by underweight status, N (%)			
Moderate to severe (WAZ <−2)	6879 (19.9)	3243 (19.5)	10 122 (19.8)
Severe (WAZ <−3)	2605 (7.5)	1238 (7.4)	3843 (7.5)

WAZ, weight-for-age z-score.

At the second measurement of WAZ, mean WAZ was −1.39 (SD=1.35) in the azithromycin arm and −1.38 (SD=1.33) in the placebo arm (table 2). There was no difference in WAZ detected (mean difference 0, 95% CI −0.05 to 0.04, p value 0.94). Sensitivity results comparing the different azithromycin arms showed similar results (table 2). Using community-level WAZ replicated these results, with a mean difference of 0.03 (95% CI −0.04 to 0.09, p value 0.44) in the azithromycin arm compared with the placebo arm (online supplemental Table 1). There was also little difference found in comparison of malnutrition subgroups, with the odds of being moderately to severely underweight in the azithromycin arm 0.99 (95% CI 0.93 to 1.06, p value 0.77) times that of those in the placebo arm, and the odds of being severely underweight in the azithromycin arm 1.03 (95% CI 0.94 to 1.13, p value 0.51) times that of those in the placebo arm (table 3).

**Table 2:** Mean difference in second instance of WAZ in infants 1–11 months with at least two weight measurements of treatment arm compared with placebo using three definitions of treatment arm

Comparison	Arm	Mean WAZ Mean, SD	Mean difference (95% CI)*	P value
Azithro versus placebo	Placebo	−1.38, 1.33	Ref	Ref
	Azithro	−1.39, 1.35	−0.002 (−0.05 to 0.04)	0.94
Azithro 1–11 m versus placebo	Placebo	−1.38, 1.33	Ref	Ref
	Azithro 1–11 m	−1.37, 1.34	0.01 (−0.05 to 0.06)	0.77
Azithro 1–59 m versus placebo	Placebo	−1.38, 1.33	Ref	Ref
	Azithro 1–59 m	−1.40, 1.36	−0.01 (−0.06 to 0.04)	0.74

* Analysis using linear mixed-effects model with random effect for village adjusting for baseline WAZ.

m, months; WAZ, weight-for-age z-score.

**Table 3:** OR of underweight status based on either moderate and severe underweight or severe underweight in infants 1–11 months old with at least two weight measurements of treatment arm compared with placebo

Comparison	Predictor	Number (%) in malnourished subgroup	OR (95% CI)*	P value
Moderate and severe underweight	Placebo	4965 (29.8)	Ref	Ref
	Azithromycin	10 412 (30.2)	0.99 (0.93 to 1.06)	0.77
Severe underweight	Placebo	1786 (10.7)	Ref	Ref
	Azithromycin	3872 (11.2)	1.03 (0.94 to 1.13)	0.51

* Analysis using generalised linear mixed model with random effect for village adjusting for baseline WAZ.

WAZ, weight-for-age z-score.

**Figure 2:**
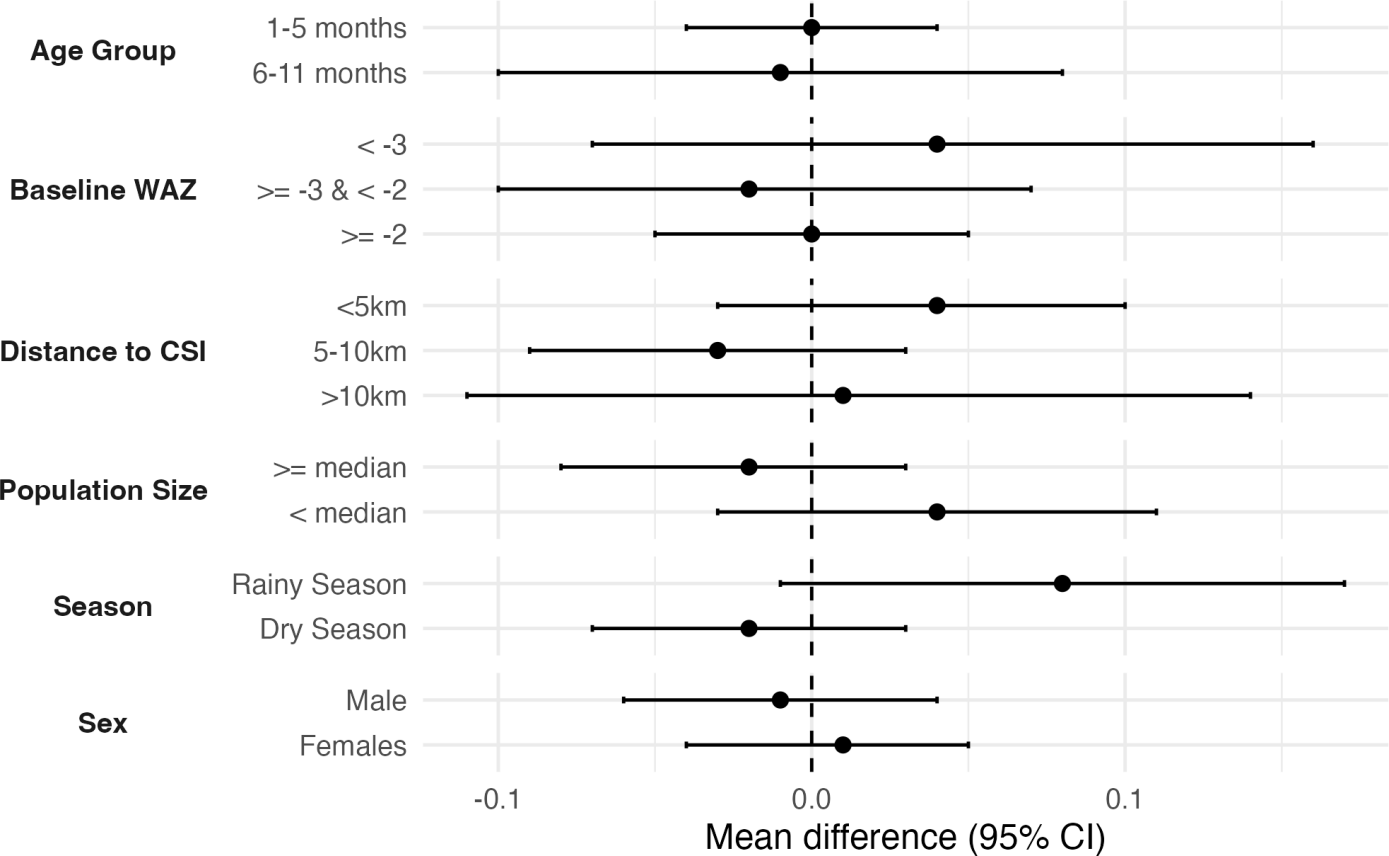
Mean difference in second instance of WAZ in infants 1–11 months with at least two weight measurements of azithromycin arm compared with placebo arm by subgroup. WAZ, weight-for-age z-score. CSI, primary health care center.

In subgroup analyses, no statistically significant differences in mean WAZ or underweight prevalence were observed (figure 2, online supplemental Table 2) by any subgroup. Effect estimates were similar by baseline nutritional status, sex and age group, with CIs generally overlapping the null. When stratified by season, infants receiving azithromycin in the rainy season had a slightly larger non-significant WAZ compared with placebo (mean difference 0.08, 95% CI −0.01 to 0.17, p value 0.08). This seasonal pattern was not evident in the dry season with a mean difference of -0.02 (95% CI −0.07 to 0.03, p value 0.42).

## Discussion

In this large, population-based cluster-randomised trial of biannual azithromycin MDA to infants aged 1–11 months in Niger, we found no evidence of a difference in WAZ or prevalence of underweight in infants receiving azithromycin compared with those receiving placebo. The lack of effect was consistent across subgroups defined by baseline nutritional status, sex, age and community size. Although no statistically significant differences were observed overall, there was a small, non-significant trend towards improved WAZ among infants treated in the rainy season that was not apparent in the dry season.

Our findings align with prior large-scale studies that have been unable to demonstrate improvements in growth following azithromycin MDA. Trials in The Gambia, Niger and Burkina Faso similarly found no sustained effects on weight or height, despite plausible biological pathways linking reduced infectious disease burden to improved nutritional outcomes.^11 14 15 21 22^ Together, these findings suggest that while azithromycin MDA can reduce all-cause mortality, it may not confer measurable medium-term benefits on weight gain in community settings. The non-significant improvement in WAZ among infants treated in the rainy season corresponds with findings from this same cohort which show that WAZ is lowest in the post-rainy season (the 3 months following the rainy season) compared with the peak dry season (6 months following the rainy season).^23^ Treatment with azithromycin during the rainy season may help decrease any loss of WAZ that seasonally occurs during the time following the rainy season. However, it is possible that this is just a chance finding given that this analysis does include multiple comparisons. Even if azithromycin does improve WAZ during the rainy season, the 0.08 increase is smaller than what would typically be considered clinically meaningful.

This study was designed to evaluate the real-world effects of MDA, rather than the effects that might be observed in tightly controlled clinical settings. While several previous studies have shown that antibiotics can improve growth when given in settings of severe acute malnutrition or other clinical scenarios,^924^,^26^ the community-based setting like that of rural Niger introduces multiple other factors that can impact growth. Food insecurity, seasonal fluctuations in food availability and high infectious disease burden likely influence growth trajectories and may attenuate any potential nutritional benefits of azithromycin.^27^,^30^ Furthermore, there is evidence that routine antibiotic use in the presence of severe acute malnutrition can improve growth outcomes in the short term; however, these benefits diminish after 8 weeks and are not sustained longer term.^31 32^ It is possible that infants in this trial experienced initial gains following azithromycin administration that had waned by the time of the 6-month follow-up assessment, consistent with what has been seen in other trials.^11^ The AVENIR trial was a community-level intervention that aimed to reduce all-cause child mortality and not directly intervene on nutrition endpoints. All nutritional interventions that are standard of care in this setting were still in place and participating in the AVENIR trial did not disqualify children of these interventions.

The mean WAZ found in the population in this analysis that incorporated infants with at least two sequential measurements was higher than that found in an analysis of the same trial population including all infants with at least only one measurement.^33^ This latter population displayed an overall greater average baseline WAZ of −1.12 (SD=0.56) with an average age of 5.6 months. Comparatively, the average WAZ in sub-Saharan Africa is −0.87, according to the most recent DHS surveys for each country.^34^ Niger has among the highest prevalence of underweight infants at 36.4%, compared with an overall prevalence of 20.1% in Western Africa.^35^ Niger holds a population of infants with a high burden of undernutrition in urgent need of intervention. Our analysis saw an average baseline WAZ of −0.8 (SD=1.5) and an average age of 3.5 months. Because the analysis required two measurements, the population skewed younger, and WAZ tends to decline in infancy with increasing age, particularly after 6 months as exclusively breastfeeding decreases.^36 37^ Thus, our results here may be biased towards younger infants with a healthier WAZ who would not be as impacted by azithromycin treatment.

Several strengths of this study enhance confidence in the findings. The AVENIR trial is one of the largest antibiotic MDA trials to date, with a population-based sample and rigorous randomisation and masking procedures. The large sample size provided sufficient power to detect small differences in WAZ and allowed for subgroup and sensitivity analyses. Weight measurements were collected using standardised methods by trained field staff. Data collection was done by a rolling census which ensured year-round data capture to assess seasonal trends.

There are also some important limitations. First, we only evaluated weight changes between two time points and did not assess shorter-term growth trajectories. It is possible that any nutritional benefits of azithromycin are acute and diminish over time. Second, we focused exclusively on infants aged 1–11 months and restricted inclusion to those with subsequent measurements who are 1–5 months old at baseline. Effects may differ in older age groups who were not part of this analysis. Third, while WAZ is a widely used indicator of nutritional status, it may not fully capture other aspects of child growth such as linear growth or body composition. Other measurements such as WHZ were not able to be included as the height stick used for dosing did not capture exact height and was not included in the data collection instrument. Finally, the large SDs of WAZ seen indicate some measurement error. In this trial, a single weight measurement was taken to determine dosing using a hanging scale, which may have resulted in some inaccuracy. Any measurement bias is expected to be non-differential between arms, thus resulting in bias towards the null.

In summary, in a large cluster-randomised trial of biannual azithromycin MDA to infants 1–11 months, we found no measurable improvement in WAZ, suggesting that azithromycin does not affect underweight status at 6 months after treatment. The suggestion that azithromycin MDA might result in higher WAZ during the rainy season merits further study, particularly given the known influence of seasonal factors on nutrition and infection. When considered alongside other large community-based trials in sub-Saharan Africa that have similarly reported no sustained effects of azithromycin MDA on anthropometric outcomes, our findings contribute to a growing body of evidence indicating that antibiotic distribution, while effective at reducing mortality, is unlikely to meaningfully improve child growth in the absence of additional interventions.

## Supplementary material

10.1136/bmjpo-2025-004037 online supplemental file 1

## Data Availability

Data are available in a public, open access repository.
