# Supplementary material for: Impact of biannual azithromycin on weight-for-age z-score among infants in the AVENIR cluster-randomised trial
Source: BMJ Paediatr Open. 2025 Dec 11;9(1):e004037. doi: 10.1136/bmjpo-2025-004037 (PMC12699659; doi:10.1136/bmjpo-2025-004037)
Supplement: online supplemental file 1 [file bmjpo-9-1-s001.docx]

**Supplemental Material**

**Supplemental Table 1.** Mean difference in second instance of WAZ at the community level in infants 1-11 months with at least one weight measurement of treatment arm compared to placebo using three definitions of treatment arm

| Comparison | Arm | Mean WAZ  Mean, SD | Mean Difference  (95% CI) | p-value |
| --- | --- | --- | --- | --- |
| Azithro vs Placebo | Placebo | -1.43, 0.88 | Ref | Ref |
|  | Azithro | -1.41, 0.85 | 0.03 (-0.04 - 0.09) | 0.44 |
| Azithro 1-11m vs Placebo | Placebo | -1.43, 0.88 | Ref | Ref |
|  | Azithro 1-11m | -1.41, 0.88 | 0.02 (-0.06 - 0.11) | 0.64 |
| Azithro 1-59m vs Placebo | Placebo | -1.43, 0.88 | Ref | Ref |
|  | Azithro 1-59m | -1.41, 0.83 | 0.03 (-0.04 - 0.1) | 0.42 |

*Analysis using linear regression adjusting for baseline WAZ

**Supplemental Table 2.** Mean difference in second instance of WAZ in infants 1-11 months with at least two weight measurements of azithromycin arm compared to placebo arm by subgroup

| Subgroup | Arm | Mean WAZ  Mean, SD | Mean Difference  (95% CI) | p-value |
| --- | --- | --- | --- | --- |
| Baseline WAZ |  |  |  |  |
| >= -2 | Placebo | -1.30 (1.30) | Ref | Ref |
|  | Azithro | -1.31 (1.33) | -0.01 (-0.05 - 0.04) | 0.84 |
| >= -3 & < -2 | Placebo | -1.64 (1.38) | Ref | Ref |
|  | Azithro | -1.65 (1.39) | -0.02 (-0.11 - 0.06) | 0.63 |
| < -3 | Placebo | -1.78, 1.41 | Ref | Ref |
|  | Azithro | -1.74 (1.46) | 0.04 (-0.08 - 0.16) | 0.55 |
| Sex |  |  |  |  |
| Males | Placebo | -1.67, 1.32 | Ref | Ref |
|  | Azithro | -1.70, 1.34 | -0.02 (-0.07 - 0.03) | 0.46 |
| Females | Placebo | -1.08, 1.27 | Ref | Ref |
|  | Azithro | -1.08, 1.30 | 0.00 (-0.05 - 0.05) | 0.97 |
| Community Size |  |  |  |  |
| < median (543) | Placebo | -1.37, 1.34 | Ref | Ref |
|  | Azithro | -1.34, 1.35 | 0.04 (-0.03 - 0.12) | 0.28 |
| >= median (543) | Placebo | -1.38, 1.32 | Ref | Ref |
|  | Azithro | -1.41, 1.36 | -0.03 -0.09 - 0.02) | 0.24 |
| Distance to CSI |  |  |  |  |
| < 5 km | Placebo | -1.42, 1.33 | Ref | Ref |
|  | Azithro | -1.40, 1.35 | 0.04 (-0.03 - 0.10) | 0.31 |
| 5-10 km | Placebo | -1.35, 1.31 | Ref | Ref |
|  | Azithro | -1.39, 1.35 | -0.03 (-0.09 - 0.03) | 0.37 |
| > 10 km | Placebo | -1.35, 1.39 | Ref | Ref |
|  | Azithro | -1.35, 1.38 | 0.01 (-0.11 - 0.14) | 0.84 |
| Age at baseline |  |  |  |  |
| 1-5 months | Placebo | -1.37, 1.32 | Ref | Ref |
|  | Azithro | -1.38, 1.35 | 0.00 (-0.05 - 0.04) | 0.85 |
| 6-11 months | Placebo | -1.43, 1.38 | Ref | Ref |
|  | Azithro | -1.46, 1.42 | -0.02 (-0.11 - 0.06) | 0.57 |
| Season |  |  |  |  |
| Rainy (July-September) | Placebo | -1.35, 1.29 | Ref | Ref |
|  | Azithro | -1.29, 1.33 | 0.08 (-0.01 - 0.17) | 0.08 |
| Dry (October-June) | Placebo | -1.39, 1.34 | Ref | Ref |
|  | Azithro | -1.41, 1.36 | -0.02 (-0.07 - 0.03) | 0.42 |

*Analysis using linear mixed effects model with random effect for village adjusting for baseline WAZ
