# Supplementary material for: Impact of biannual azithromycin on weight-for-age z-score among infants in the AVENIR cluster-randomised trial
Source: BMJ Paediatr Open. 2025 Dec 11;9(1):e004037. doi: 10.1136/bmjpo-2025-004037 (PMC12699659; doi:10.1136/bmjpo-2025-004037)
Supplement: Supplementary file 2 [file bmjpo-2025-004037.reviewer_comments.pdf]

PEER REVIEW HISTORY

BMJ Paediatrics Open publishes all reviews undertaken for accepted manuscripts. Reviewers are asked to complete a checklist review form and are provided with free text boxes to elaborate on their assessment. These free text comments are reproduced below.

ARTICLE DETAILS

**TITLE (PROVISIONAL)**	The impact of biannual azithromycin on weight-for-age z-score among infants in the AVENIR cluster-randomized trial
**AUTHORS**	Peterson, Brittany; Arzika, Ahmed; Maliki, Ramatou; Abdou, Amza; Aichatou, Bawa; Bello, Ismael; Beidi, Diallo; Galo, Nasser; Harouna, Nasser; Karamba, Alio; Mahamadou, Sani; Abarchi, Moustapha; Ibrahim, Almou; Lebas, Elodie; Liu, Zijun; Brandt, Carolyn; Colby, Emily; Oldenburg, Catherine; Porco, Travis; Arnold, Benjamin; Lietman, Thomas; O'brien, Kieran S

VERSION 1 - REVIEW

**REVIEWER NAME**	Nina Paynter
**REVIEWER AFFILIATION**	None disclosed
**REVIEWER CONFLICT OF INTEREST**	N/A
**AI DISCLOSURE**	No
**DATE REVIEW RETURNED**	28-Sep-2025

**GENERAL COMMENTS**	This is a throughly explained and well-written analysis using a large clinical trial to assess a secondary endpoint with clear methods and results. I have no additional comments or requests for clarification prior to publication.

**REVIEWER NAME**	Vandana Prasad
**REVIEWER AFFILIATION**	Public Health Resource Society, New Delhir
**REVIEWER CONFLICT OF INTEREST**	N/A
**AI DISCLOSURE**	No
**DATE REVIEW RETURNED**	22-Oct-2025

**GENERAL COMMENTS**	This is a really neat and helpful study. A few clarifications and suggestions, as below might be addressed for greater value to the readers.<br><br>1. Why was WHZ not considered as the primary outcome for this particular study, and that could be discussed in the discussion section. I presume lengths were not done for infants.<br>2. Why were heights done for 12-59 months for dosage rather weights, considering weights are considered easier, weights were done for the intervention group and weight for age was the primary outcome for the study?<br>3. Were the heights of 12-24 months converted to lengths? (the latter 2 questions are relevant for people studying methods for malnutrition work)<br>4. As an interventionist one would like to have some sense of whether programmes are currently using antibiotics as community

	level interventions, and whether there is a trade off or opportunity cost to this in terms of compromises on direct nutritional interventions.<br>5. Just as an aside as a member of the public health nutrition community, in our 15 year long MIS data, we see our maximum deteriorations at ages 6 to 11 months despite a fairly intense intervention.

VERSION 1 – AUTHOR RESPONSE

Reviewer: 1

Dr. Nina Paynter

Comments to the Author

This is a thoroughly explained and well-written analysis using a large clinical trial to assess a secondary endpoint with clear methods and results. I have no additional comments or requests for clarification prior to publication.

Response: Thank you for your comment and we appreciate your review of the manuscript.

Reviewer: 2

Dr. Vandana Prasad, Public Health Resource Society, New Delhir

Comments to the Author

This is a really neat and helpful study. A few clarifications and suggestions, as below might be addressed for greater value to the readers.

1. Why was WHZ not considered as the primary outcome for this particular study, and that could be discussed in the discussion section. I presume lengths were not done for infants.

Response: Thanks for this question. While WHZ would be an interesting endpoint to study as well, height was not captured as part of data collection. A height stick was used for dosing which did not capture exact height and was not included in the data collection instrument. We have included this as part of the limitations in the discussion section on lines 386-388.

2. Why were heights done for 12-59 months for dosage rather weights, considering weights are considered easier, weights were done for the intervention group and weight for age was the primary outcome for the study?

Response: Great question. In this study, treatment occurred door-to-door in the study communities and the hanging-scale used for weighing is logistically more complicated to use at the participants houses, so weighing was only used for the necessary age group of 1-11 months as they are least likely to be able to stand. Height-dosing sticks were used for children 12-59 which indicates doses that are based on height ranges, but does not indicate specific heights. We've included information about this in the methods section under the intervention subheading, lines 228-231.

3. Were the heights of 12-24 months converted to lengths? (the latter 2 questions are relevant for people studying methods for malnutrition work)

Response: Since the dosing pole was not used to capture height, but rather to calculate dosage heights were not captured and therefore not converted to lengths. As noted above, the dosing pole indicates doses that are based on height ranges, but does not indicate specific heights. We've added clarification about this in the methods section, data collection subheading, lines 220-222.

4. As an interventionist one would like to have some sense of whether programmes are currently using antibiotics as community level interventions, and whether there is a trade off or opportunity cost to this in terms of compromises on direct nutritional interventions.

Response: This is a great comment. The AVENIR trial was a community-level intervention that aimed to reduce all-cause child mortality and was not directly intervening on nutrition endpoints. All nutritional interventions that are standard of care in this setting were still in place and participating in the AVENIR trial did not disqualify children of these interventions. We've added this information in the discussion section, lines 357-360.

5. Just as an aside as a member of the public health nutrition community, in our 15 year long MIS data, we see our maximum deteriorations at ages 6 to 11 months despite a fairly intense intervention.

Response: Great insight. We've acknowledged in the discussion that WAZ tends to decline in infancy with increasing age, particularly after 6 months as exclusively breastfeeding decreases, lines 371-372.
